# Complex molecular mechanisms underlying MYMIV-resistance in *Vigna mungo* revealed by comparative transcriptome profiling

**DOI:** 10.1038/s41598-019-45383-w

**Published:** 2019-06-20

**Authors:** Anirban Kundu, Pankaj Kumar Singh, Avishek Dey, Sayak Ganguli, Amita Pal

**Affiliations:** 1 Division of Plant Biology, Bose Institute, Kolkata, 700054 India; 2 Ramakrishna Mission Vivekananda Centenary College, Rahara, Kolkata, 7000118 India; 3 Theoretical and Computational Biology, AIIST, Palta Kolkata, India

**Keywords:** Transcriptomics, Biotic

## Abstract

Mungbean Yellow Mosaic India Virus (MYMIV)-infection creates major hindrance in *V*. *mungo* cultivation and poses significant threat to other grain legume production. Symptoms associated include severe patho-physiological alterations characterized by chlorotic foliar lesion accompanied by reduced growth. However, dissection of the host’s defense machinery remains a tough challenge due to limited of host’s genomic resources. A comparative RNA-Seq transcriptomes of resistant (VM84) and susceptible (T9) plants was carried out to identify genes potentially involved in *V*. *mungo* resistance against MYMIV. Distinct gene expression landscapes were observed in VM84 and T9 with 2158 and 1679 differentially expressed genes (DEGs), respectively. Transcriptomic responses in VM84 reflect a prompt and intense immune reaction demonstrating an efficient pathogen surveillance leading to activation of basal and induced immune responses. Functional analysis of the altered DEGs identified multiple regulatory pathways to be activated or repressed over time. Up-regulation of DEGs including NB-LRR, WRKY33, ankyrin, argonaute and NAC transcription factor revealed an insight on their potential roles in MYMIV-resistance; and qPCR validation shows a propensity of their accumulation in VM84. Analyses of the current RNA-Seq dataset contribute immensely to decipher molecular responses that underlie MYMIV-resistance and will aid in the improvement strategy of *V*. *mungo* and other legumes through comparative functional genomics.

## Introduction

*Vigna mungo* (blackgram) is one of the most consumed grain legumes, cultivated mostly by the Asiatic populations for food and medicinal properties promoting nutritional benefits for human health^1,2^. Owing to its ability of symbiotic nitrogen fixation, blackgram seeds serve as a vital source of dietary protein. However, predominance of the crop is threatened by various pests and pathogens that affect both quality and productivity. Yellow mosaic disease (YMD) has the most detrimental effect in productivity, imposing a significant yield loss with estimates of up to 100% under conducive environments^3,4^. This prodigious economic loss is predominantly caused by the bipartite begomovirus, Mungbean Yellow Mosaic India Virus (MYMIV), the etiological agent of YMD. As an obligate whitefly-borne pathogen, MYMIV has an extensive natural host-range that includes several legume species and intensifies yield reduction primarily upsetting the physiological machinery of the host^5^.

Presently, host’s genetic resistance is the most preferred management strategy in mitigating crop loss^6,7^. This relies largely on the interaction of host-specific resistance (R) proteins and their cognate pathogenic avirulence (Avr) effectors^8,9^. Such interactions are governed by an array of genes activated over time and inducing signal transduction cascades that decide the fate of the host. However, presence of a defunct allele of the R-gene resulted in a compatible interaction influencing pathogen colonization^10^. Seven natural mutants derived from *V*. *mungo* Cv. T9 were selected that demonstrated long-lasting genetic resistance to MYMIV^3^. Subsequent study has shown that MYMIV-resistance is conferred by the R-gene, *CYR1* encoding a NBS-LRR protein^11^. Despite its agrarian importance, limited studies have been attempted to decipher the molecular mechanisms that underlie response of *V*. *mungo* against MYMIV. But, undoubtedly YMD management in the long-run will rely on the improvements in genomics-assisted breeding.

In this study we have undertaken a comparative transcriptome analysis through RNA-Seq to unravel the key molecular players of MYMIV-resistance in this orphan crop. Genome wide transcriptome analysis is a powerful tool to understand the molecular responses of the host during the pathogen invasion. Procedure of exploring the molecular basis of the MYMIV-stress response in *V*. *mungo* largely relies on the transcriptomic differences between the resistant and susceptible genotypes, prior to and after pathogen-inoculation. Previously, 345 differentially expressed ESTs were identified in *Vigna* upon MYMIV inoculation by suppression subtractive hybridization^5^. A robust induction of defense signaling underlined the resistant reaction, while a feeble expression of the defense genes were recorded in the susceptible genotype that got attenuated with time. Additionally, a comprehensive proteomics data of two contrasting genotypes across a three week infection period have complemented the SSH data^12^. Despite such insights on the host-pathogen interactions, the understanding of defense mechanism remained incomprehensible; since only a subset of genes were analyzed in relation to the complex patho-physiology of the interaction. Therefore, we had envisaged that perhaps a genome wide analysis of the host transcriptome may bridge the prevailing lacunae and augment our current knowledge of the complex interaction and influence the outcome of infection.

Based on the above backdrop, experiments were designed to identify and correlate the differential expression of genes on a global scale from mock and MYMIV-inoculated resistant and susceptible genotypes. RNA-Seq offers an affordable and accessible technology that enables rapid acquisition of high resolution sequence datasets, offering a precise assessment of gene expression levels^13,14^. This technology is of particular interest in analyzing the global transcriptional networks in non-model organisms, primarily in those lacking the genome-sequence information. Genome-wide expression profiles during the different stages of host-pathogen interaction would eventually aid in mining of candidate transcripts that could be employed for designing plant-targeted control strategies against the viral pathogen. Unfortunately, little progress has been made in this regard for this legume-crop. The present work exemplifies a comprehensive scenario of transcripts reprogramming and pinpoints the key molecular determinants associated with defense signaling, host physiology and metabolism. Indeed, the outcome of this study will unravel the complex molecular architecture of the MYMIV-*V*. *mungo* pathosystem that precedes genomic assisted breeding.

## Results

### MYMIV infectivity assays in susceptible and resistant genotypes

**Figure 1:**
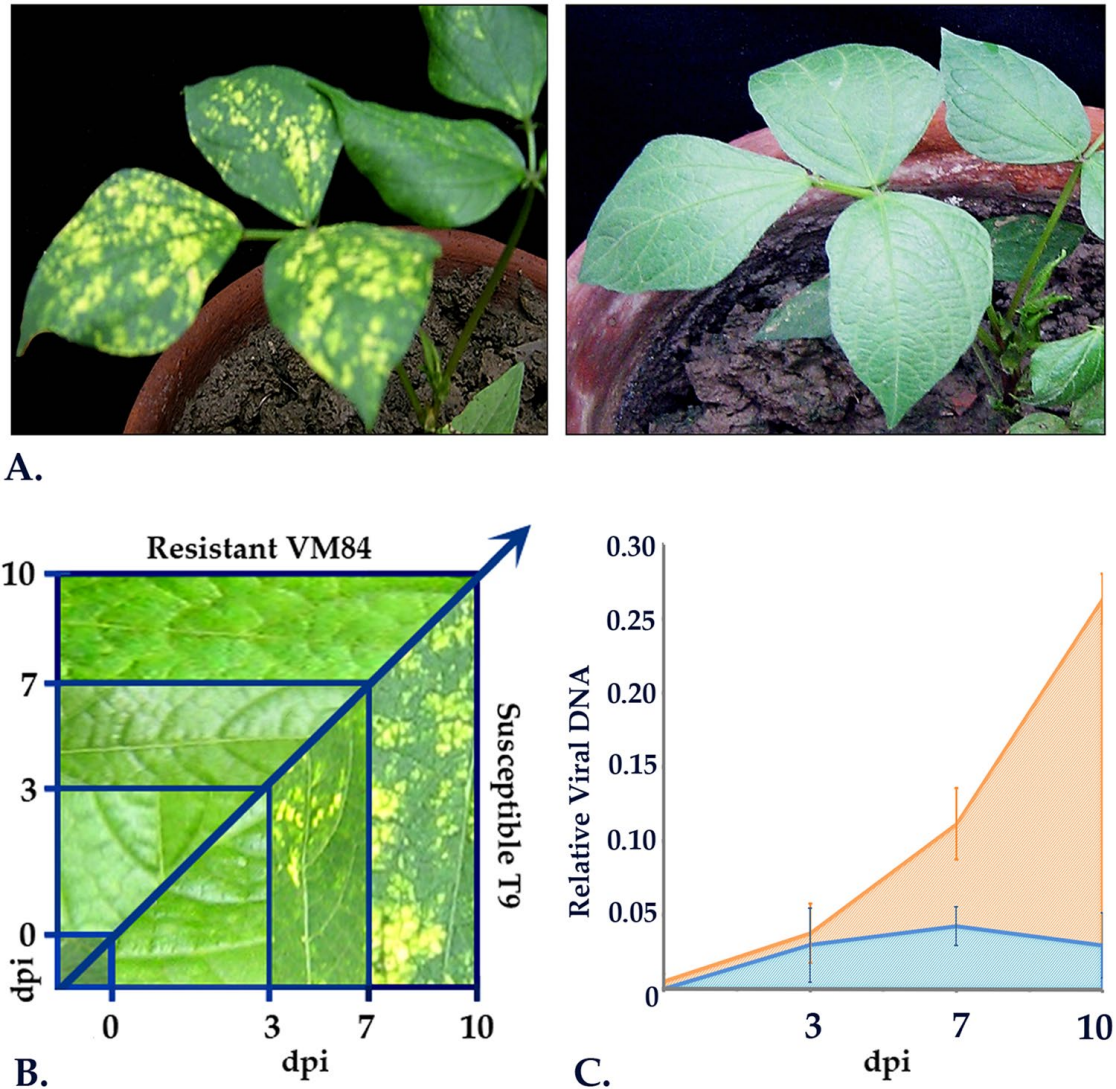
MYMIV infestation in resistant and susceptible *V*. *mungo*. (**A**) Post inoculation phenotype in susceptible and resistant *V*. *mungo* plants at 10 dpi. (**B**) Time course analysis of disease progression in susceptible (T9) and resistant (VM84) genotypes. (**C**) qPCR detection of MYMIV titer in susceptible and resistant *V*. *mungo* plants using MYMIV-cp DNA specific primers.

Three-week-old healthy *V*. *mungo* plants at the first trifoliate stage were challenged with viruliferous whiteflies as treated samples (MI) and with aviruliferous whiteflies as mock-inoculated (MC) samples. Infectivity was analyzed following inoculations and symptoms development was monitored every 24 h and compared to the mock-inoculated plants (Fig. 1A). Along with *in planta* symptom development, multiplications of MYMIV were also recorded using qPCR. During the early stages of infection, all the plants remained asymptomatic up to 3 days following which initial signs of mosaics started to appear in susceptible T9 (Fig. 1B). With the progression of incubation time, an increasing number of yellow specks with irregular margins gradually appeared and lesions spread rapidly from 7 days post inoculation (dpi). Yellow mosaic spots started to coalesce 10 dpi onwards forming larger specks and at 15 dpi the inoculated leaves turned fully symptomatic showing mild leaf distortions. In contrast, the MYMIV-inoculated-resistant and all the mock-inoculated plants remained symptom-free.

Consequently, three sampling-points, one at pre-symptomatic stage viz. 3 dpi, followed by an intermediate (7 dpi) and at later stages of infection (10 dpi) were chosen for RNA-Seq and qPCR analysis (Fig. 1C). Trifoliate leaves from the same inoculated plants were further processed for qPCR analyses to measure the extent of virus-pathogen proliferation. Copy number of the MYMIV-coat-protein (cp) gene was calculated for 3 biological replicates and represented by mean values obtained at each sampling point. Assuming the quantity of cp gene correlates with the degree of infection, amplification of the cp gene was observed exclusively in the MYMIV-inoculated susceptible plants as anticipated. Although, MYMIV-cp gene was detected in the resistant background, the fold change observed were too miniscule to be considered significant, and presumably represent the results of initial replication following MYMIV-inoculation. Moreover, the post MYMIV-inoculation alterations in gene expression in resistant and susceptible genotypes were normalized against the mock-inoculated controls to ensure that the changes are solely due to the viral pathogen.

### Transcriptome profiling and assembly of reads

IlluminaTruSeq transcriptome profiling was conducted from both resistant (VM84) and susceptible (T9) genotypes that resulted in the generation of 4 libraries (resistant: 84MC, 84MI and susceptible: T9MC and T9MI). To avoid sample bias, 3 samples were collected at three different time points each of VM84 and T9 post-MYMIV- and mock-inoculations, processed independently for RNA isolation and pooled in equimolar concentration for the construction of the RNA-Seq libraries. A total of about 1,44,234,850 bp (T9 MC: 73,322,419 bp and T9 MI: 70,912,43 bp) and 1,84,815,786 bp (84MC: 77,342,016 bp and 84 MI: 1,07,473,770 bp) raw 101 bp paired end reads were generated from T9 and VM84 libraries, respectively. Subsequently, after quality filtering, a leftover of 1,40,278,890 bp and 1,74,911,047 bp clean reads were obtained from T9 and VM84 libraries, respectively. *De novo* assembly of the clean reads generated 46,011, 43,941, 49,720 and 1,03,842 transcripts for T9MC, T9MI, 84MC and 84MI, respectively. Average length of transcripts for T9 and VM84 are 1529.7 and 1531.7 bp, respectively. Other parameters like number of transcripts ≥500 bp, ≥1 Kbp, N50 size and GC content (%) for each of the 4 individual libraries are presented in Table 1. Quality passed raw reads of all the four analyzed libraries were deposited in National Centre for Biotechnology Information (NCBI) Sequence Read Archive (SRA) under the accession numbers SRX1032950 (84MC), SRX1082731 (84MI), SRX1058325 (T9MC) and SRX1058327 (T9MI).

**Table 1:** Statistics of the *de novo* assembled *V*. *mungo* [MYMIV-inoculated (MI) and mock-inoculated (MC)] transcriptomes.

Parameters	Susceptible		Resistant	
	T9MC	T9MI	84MC	84MI
Total number of raw reads	73,322,419	70,912,431	77,342,016	1,07,473,770
Number of reads used	72,124,101	68,154,789	74,615,918	1,00,295,129
Transcripts generated	46,011	43,941	49,720	1,03,842
Maximum transcript length (bp)	15,601	15,585	15,357	19,652
Minimum transcript length (bp)	200	200	200	200
Average transcript length (bp)	1543.2	1516.2	1688.2	1375.2
Transcripts ≥500 bp	41,785	39,532	42,048	76,066
Transcripts ≥1 Kbp	25,558	26,794	33,281	54,945
N50 size (bp)	1,928	1,893	2,254	2,031
GC content (%)	39.57	39.71	40.96	41.3

**Figure 2:**
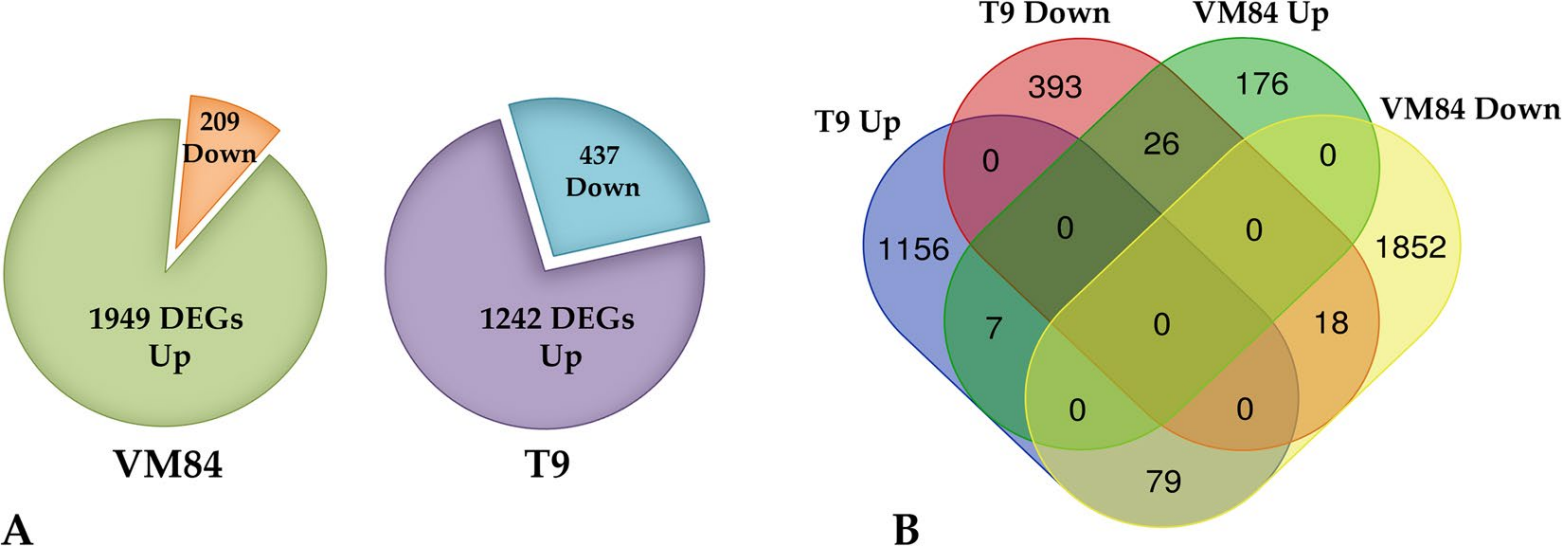
Comparison of differentially expressed genes (DEGs) in resistant and susceptible *V*. *mungo* plants: (**A**) Venn diagram illustrating the share of up and down regulated DEGs of *V*. *mungo* in response to MYMIV, (**B**) Comparison of DEGs depicting the share of overlapped and non-overlapped transcripts in susceptible (T9) and resistant (VM84) hosts.

Transcriptome profiles of these two contrasting genotypes varied considerably depending on the responses mounted against the pathogen with greater modulation in the resistant background than the susceptible one, showing clear evidence of correlation between expressional alteration and immune response. Using the DESeq statistical package, differentially expressed transcripts were considered significant that exhibited P-value ≤ 0.05 and a Log 2 Ratio >2.0. Applying this threshold, comparison between mock and MYMIV-inoculated VM84 revealed 1949 transcripts with induced and 209 with repressed expression, totaling 2158 differentially expressed genes (DEGs). While susceptible T9 responded through 1679 DEGs, 1242 of which was up-regulated and 437 down-regulated in the experimental conditions analyzed (Fig. 2A). Proportions of unique and shared DEGs among the contrasting genotypes are portrayed in the Venn diagram (Fig. 2B).

### Functional enrichment of *V*. *mungo* DEGs in response to MYMIV

**Figure 3:**
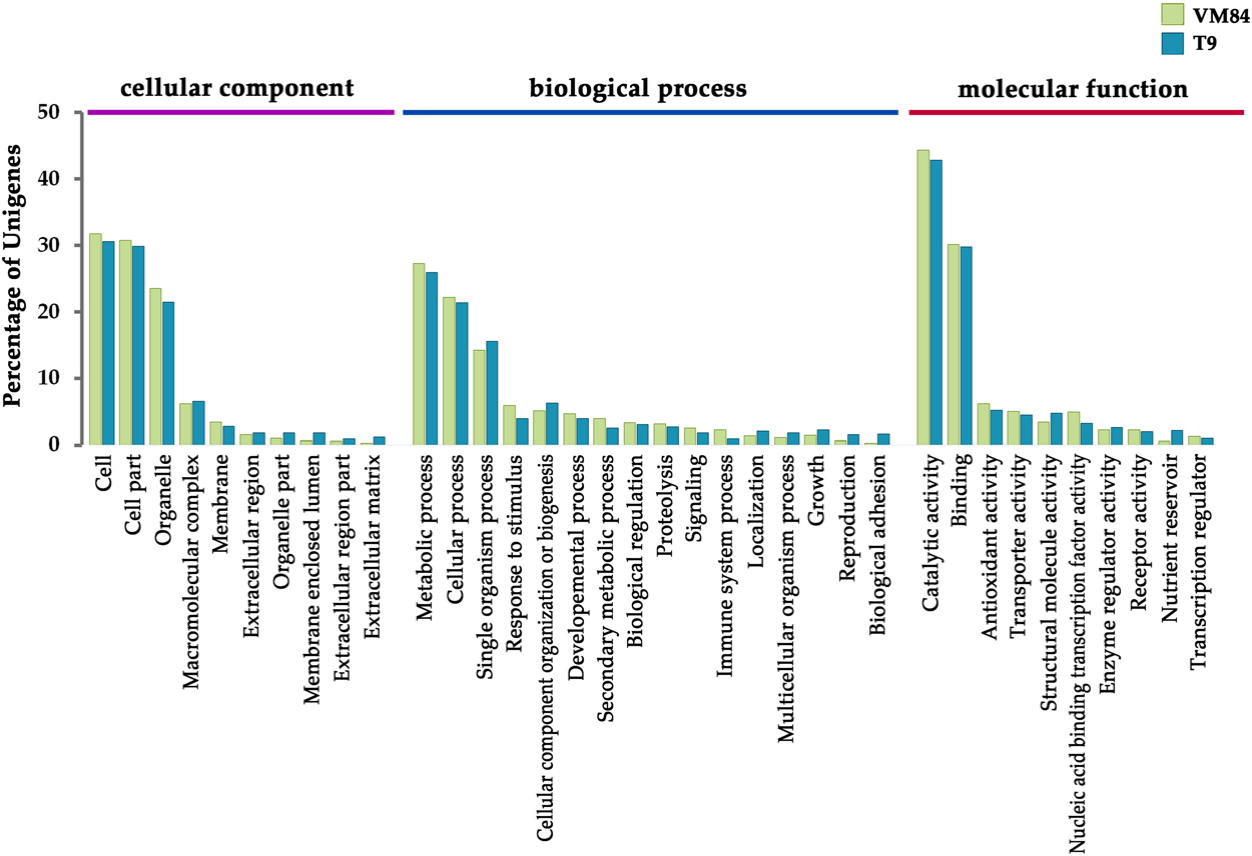
Gene ontology (GO) classification of the differentially expressed genes (DEGs) in *V*. *mungo*. DEGs were classified in three main GO categories of biological process, cellular component and molecular function. The Y-axis indicates percentage of unigenes.

Gene ontology (GO) analysis using the Blast2GO software resulted in the identification of the major functional groups and revealed biological significance of the DEGs involved in MYMIV-response in coordinate relation to three GO terms viz. cellular component, biological process and molecular function (Fig. 3). Amongst the cellular components, the major GO terms related to cell, cell part and organelle were found to be enriched upon MYMIV inoculation, while the other categories were represented weakly. Concerning the biological process, the most represented categories include “metabolic process” (GO:0008152) and “cellular process” (GO:0009987) followed by “response to stimulus” (GO:0050896), “signaling” (GO:0023052) and “immune system process” (GO: 0002376) were found to be reprogrammed in both susceptible and resistant backgrounds. Noteworthy enrichment was also noted in some interesting categories such as “secondary metabolic process” (GO:0019748), “biological regulation” (GO:0065007) and “proteolysis” (GO:0006508), the components of which may play important roles in determining the host’s reaction against MYMIV. Despite having several common GO terms shared by the both the resistant and susceptible genotypes, yet a substantial heterogeneity was noted in the contributing DEG pool with or without any overlapping genes, thereby displaying distinct reactions to MYMIV.

Identified DEGs were mapped to the KEGG database using the Blast2GO platform to gain an insight into the major metabolic pathways operating in response to MYMIV. Pathway enrichment analysis assigned KEGG orthologous number to 2756 DEGs and mapped them in 97 metabolic pathways. Five highly represented groups include “metabolic pathways” (ko01100), “biosynthesis of secondary metabolites” (ko01110), “plant pathogen interaction” (ko04626,), “plant hormone signal transduction” (ko04075) and “starch and sucrose metabolism” (ko00500). A summary of the inter-group analysis in between pathogen inoculated resistant (VM84) and susceptible (T9) revealed 79 common pathways showing significant enrichment in 11 pathways (Table 2). Greater enrichment of the “metabolic pathway” group in the susceptible (32.09%) than in resistant (26.76%) suggests that MYMIV exploits the host metabolism during pathogenesis. DEGs encoding core components of the “plant pathogen interaction” (10.29%) and “biosynthesis of secondary metabolites” (10.23%) in VM84 were assumed to implement a strong immune reaction against MYMIV. Moreover, enrichment of “Plant hormone signal transduction” (9.17% in VM84 and 6.42% in T9) and “Starch and sucrose metabolism” (5.58% in VM84 and 3.41% in T9) were also substantially higher in the resistant host. In addition to the above categories, 3.35% and 1.72% of DEGs were classified in “phenyl-propanoid biosynthesis”, 3.18% and 2.34% in “flavonoid biosynthesis” followed by 3.93% and 4.49% in “photosynthesis” in VM84 and T9, respectively. Overall, KEGG analysis of the DEGs revealed pathways that are involved in immune responses of *V*. *mungo* against MYMIV.

**Table 2:** List of top 11 metabolic pathways as revealed by KEGG enrichment analysis.

Sl. No.	KEGG Pathways	Pathway ID	% of DEGs	
			VM84*	T9**
1.	Metabolic pathways	ko01100	26.76	32.09
2.	Biosynthesis of secondary metabolites	ko01110	10.23	7.66
3.	Plant pathogen interaction	ko04626	10.29	4.45
4.	Plant hormone signal transduction	ko04075	9.17	6.42
5.	Starch and sucrose metabolism	ko00500	5.58	3.41
6.	Photosynthesis	ko00195	3.93	4.49
7.	Phenylpropanoid biosynthesis	ko00940	3.35	1.72
8.	Flavonoid biosynthesis	ko00941	3.18	2.34
9.	Ribosome	ko03010	2.42	1.50
10.	Biosynthesis of amino acids	ko00616	2.26	1.34
11.	Fatty acid metabolism	ko10703	3.43	1.65

^*^Resistant; **Susceptible.

**Figure 4:**
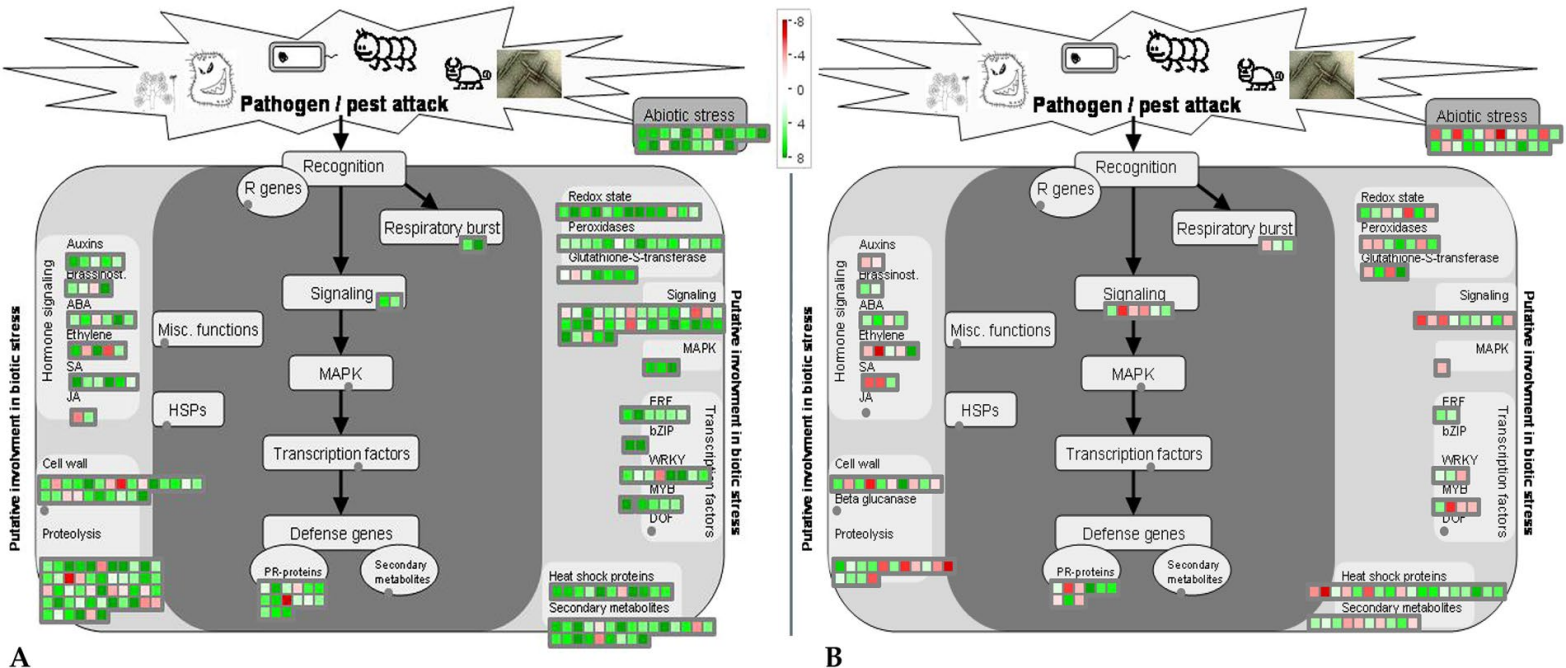
MapMan overview of MYMIV-*V*. *mungo* biotic stress pathways: Schematic representation of the DEGs involved in resistant genotype (**A**) and susceptible *V*. *mungo* genotype (**B**) in response of MYMIV infection and grouped in MapMan bins under biotic stress pathways. Green and red boxes represent the up-regulated and down-regulated genes, respectively upon MYMIV-infection. Magnitude of gene expression is associated with a gradient of colour change that represented the log fold ratios. Fold change scale of expression is inserted in the figure.

An additional level of analysis was carried out using the MapMan annotation tool to attain an in-depth understanding of the biotic stress pathways that are modulated in response to MYMIV. A comprehensive classification of the DEGs with a Log2 fold change ≥1 or ≤−1 (adjusted p ≤ 0.05) mapped 272 DEGs in VM84 and 147 in T9 in putative pathways related to biotic stress (Fig. 4). Inspection of the biotic stress pathways revealed not only a higher accumulation of immune responsive transcripts in resistant VM84 but also overall repression of these MYMIV responsive genes in susceptible T9. Greater induction of reactive oxygen species (ROS) scavenging enzymes (glutathione S transferase, peroxidases etc.) was observed in VM84 compared to T9 that detoxifies superoxide and hydroxyl radicals produced by plants to cease pathogen invasion. The present study also identified several over-expressed pathogenesis related (PR) genes including the members of the family PR1, PR5 and PR10 that act synergistically to generate of systemic acquired resistance (SAR) in distal parts of the plant. In the resistant host, 50 genes were allotted to MapMan bins designated as “Proteolysis”, followed by 35 in “signaling” and 24 genes in “secondary metabolites”, while these numbers were poorly represented with either weaker induction or suppression in the susceptible plant. MYMIV-inoculated resistant plants transcribe a plethora of genes that participate in defense signaling, encoding proteins, of which receptor like kinases (RLKs), MAP kinases (MAPKs), serine threonine kinases (STKs) are of particular interest. While other over-represented categories include transcription factors and heat shock proteins. DEGs including several members of WRKY, MYB, bHLH were up-regulated in the resistant host. Expression profiles indicate stimulation of SA levels in inoculated tissues, accompanied by the up-regulation of auxin and ethylene responsive genes. As expected, major fraction of the DEGs showed abundant and unique expression related to the above mentioned bins during the immune reaction.

**Figure 5:**
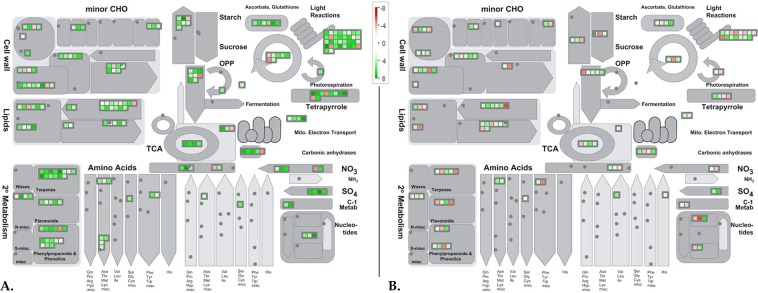
MapMan overview of MYMIV-*V*. *mungo* metabolic pathways: Schematic representation of the DEGs involved in resistant- (**A**) and susceptible- (**B**) *V*. *mungo* genotypes in response of MYMIV-infection and grouped in MapMan bins of metabolic pathways. Green and red marks represent the up- and down-regulated genes, respectively upon MYMIV-infection. Magnitude of gene expression is associated with a gradient of colour change that represented the log fold ratios. Fold change scale of expression is inserted in the figure.

Global analyses of the MYMIV-responsive transcriptome revealed an outline of the physiological alterations initiated by the viral pathogen and mapped a substantial share of genes in metabolic pathways, and allocations were identified using the MapMan tool (Fig. 5). Widespread induction of transcripts was recorded for the genes involved in various components of photosynthetic apparatus. Genes encoding proteins of PSI and PSII, chl a/b binding and others implicated in light dependent pathways were induced in the resistant background. In contrast, as per their expression profile, large majority of transcripts were found to be repressed under this category in the susceptible genotype following infection. Several candidates involved in calvin cycle (4 genes), chlorophyll biosynthesis (8 genes) and cellular respiration (15 genes) were also up-regulated in MYMIV-inoculated VM84. MapMan image also showed alteration of various genes encoding cell wall modifying proteins including cellulose synthase, xyloglucanendo-transglycosylase, pectin esterase that act concurrently to strengthen the cell wall. Genes associated with lipid metabolism were similarly over-expressed indicating their deployment as the alternative sources of energy to counter pathogen ingression. Similar induction was also recorded for enzymes participating in β-oxidation pathway that produces energy by breaking fatty acids molecules. However, as a consequence of MYMIV-infection, several transcripts related to these pathways were repressed in susceptible background. Besides instigation of the primary metabolic pathways in the resistant genotype, VM84 appear to allot carbon and nitrogen towards synthesis of secondary metabolites. Several genes, associated with the production of phenylpropanoids, phenolics, flavonoids and terpenoids, were strongly induced upon pathogen inoculation. Furthermore, expression of 3 genes involved in lignin biosynthesis indicates active deposition of lignin offering required arsenal to restrict MYMIV proliferation. Altogether, results of the GO, KEGG and MapMan analyses are categorically harmonizing and reflect an impression of the pathways that are operative in providing immune reaction to MYMIV and provide a plausible explanation for the difference in MYMIV-response between VM84 and T9.

### Validation of RNA-Seq by qRT-PCR

**Figure 6:**
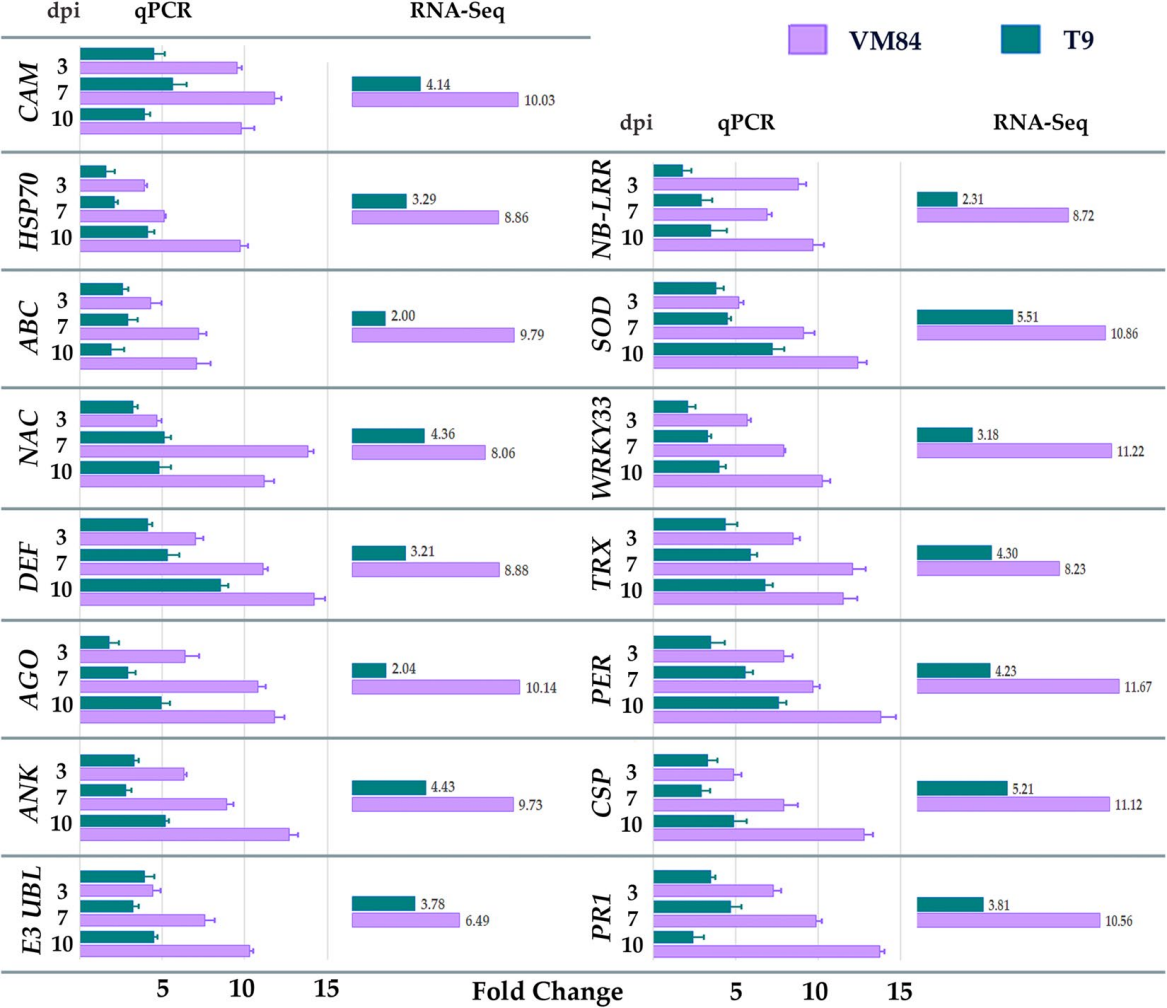
Comparative expression profiles of DEGs as revealed by qPCR with that of RNA-Seq data. qPCR expression profiles of DEGs at 3, 7, 10 days post inoculation (dpi) selected for their plausible immune responsive roles in the resistant *V*. *mungo* genotypes against MYMIV and compared with their RNA-Seq expression data. Histogram represents the expression level of each transcript while the error bars represents the standard deviation in relative expression profiles of three biological replicates. Full forms of the abbreviated genes are: *CAM*, Calmodulin; *HSP70*, Heat shock protein 70; *ABC*, ABC transporter; *NAC*, NAC domain transcription factor; *DEF*, Defensin; *AGO*, Argonaute; *ANK*, Ankyrin; *E3 UBL*, E3 Ubiquitin ligase; *NB-LRR*, Nucleotide binding –Leucine rich repeat; *SOD*, Superoxide dismutase; *WRKY33*, WRKY DNA-binding protein 33; *TRX*, Thioredoxin; *PER*, Peroxiredoxin; *CSP*, Cysteine protease; *PR1*, Pathogenesis related protein 1.

Real-time PCR (qPCR) was conducted to verify the expression profiles of some candidate genes as identified by the Illumina RNA-Seq transcriptome data. Fifteen *V*. *mungo* transcripts of potential interest, showing differential expression either in MYMIV-resistant and susceptible hosts or in both, were selected on the basis of their anticipated roles as immune mediators (Fig. 6). Amongst the selected DEGs, five (PER, TRX, DEF, ANK and SOD) were previously reported to be involved in host’s defense response and showed significant up-regulation compared to those in mock-inoculated plants. Analysis of 2 TFs, NAC (12 fold in VM84 and 5 fold in T9) and WRKY33 (10 fold in VM84 and 4 fold in T9) exhibited an enhanced expression profile at 10 dpi in the resistant than in the susceptible host and is in good accordance with the RNA-Seq data. Alteration of transcripts predicted to be involved in defense signaling is represented by NB-LRR and CAM, both of which were shown to be up-regulated at each of the sampled time points in the resistant compared to the susceptible host. Expression of other DEGs encoding ABC transporter, AGO and CSP were also confirmed to be relatively higher in all the time points of the resistant host showing slight deviation with the RNA-Seq fold change. However, expression level of HSP70 in susceptible background fluctuated from the corresponding RNA-Seq data, showing a 4 fold qPCR-accumulation at 10 dpi. This inconsistent profile may have resulted due to the dynamic nature of the transcriptome. Altogether, incompatible interaction was characterized by highly induced expression in most of the analyzed DEGs in between 7–10 dpi. This period of highest expression of immune responsive molecular components in the resistant host also corresponds to the restricted accumulation of these transcripts in the susceptible plants conforming the period of virus proliferation and symptoms development. Thus the qPCR results showed high consistency with the RNA-Seq data and established the reliability and reproducibility of transcriptome sequencing performed in the current study.

## Discussion

Upgrading the preliminary knowledge of MYMIV-induced transcriptomic responses in *V*. *mungo* is imperative to understand the molecular mechanisms for resistance and susceptibility. MYMIV, being an obligate biotrophic pathogen, developed exquisite mechanisms to interfere with host processes that can lead to several fundamental changes inducing a massive alteration in the host transcriptome. While, rapid activation immune-responders mounts an effective immune-response in the resistant host after pathogen invasion which was otherwise latent in absence of the pathogen. Therefore, an RNA-Seq facilitated-transcriptome profiling was warranted to unravel the true complexity of this interaction in a global scale which has now became comprehensible.

Hosts response to stresses relies on the timely recognition of the intruder there by integrating external cues into own cellular and physiological machinery. It was confirmed earlier that the MYMIV-susceptible T9 carried a defunct allele of the resistant gene resulted in a defective immune system^10^. Our previous study, which involved proteomic and EST analyses^5,12^ showed significantly high expression of pathogen responsive genes in the resistant host that showed delayed-expression in the susceptible host. However, those studies surveyed only a subset of genes that restricts our understanding beyond which the mechanism of MYMIV-resistance remained obscured. The present study was therefore conducted with a working hypothesis to identify the molecular determinants on a global scale along the course of infection to monitor the differential transcript re-programming between compatible and incompatible hosts that was triggered upon MYMIV-inoculation.

A comparative analysis of the four RNA-Seq libraries provided a comprehensive outline of the highly complex network encompassing the various stages of infection and allowed detection of more than 10,000 DEGs. The considerably large dataset when subjected to pathway enrichment analysis identified the DEG’s to be operative in and about 97 pathways and led us to better comprehend the complex immune-response of *V*. *mungo* to MYMIV-infestation. Virus titers were quantified in the inoculated trifoliates at three time points encompassing pre-symptomatic, symptomatic and late infection phases to confirm that our dataset comprised of all the stages of viral pathogenesis that correlated well with the symptom development. Additionally, it was also evident that the resistant host responded to MYMIV-inoculation by an instant burst of gene expression when equated with the susceptible host. GO enrichment analysis highlighted “response to stimulus” as a prominent category during the incompatible interaction that remain enriched with defense related genes in contrast to compatible reaction. While, the data revealed a waning trend of immunity during in course of disease progression in the susceptible background. All these results clearly demonstrated the differences of the underlying molecular mechanisms of these two contrasting genotypes and their differential responses against the pathogen by re-programming physiological and metabolic activities either in order to mount an immune response or succumb to the pathogen aggression.

The interplay between pathogen sensing and immune signaling could lead to the significant interventions defining the consequence of pathogen inoculation. Expression profiles of several pattern triggered immunity (PTI)-related genes including receptor-like kinases (RLKs), serine threonine protein kinase (STPK), cysteine rich receptor-like protein kinase, BED finger in MYMIV-resistant background were up-regulated relative to susceptible host. Since PTI-responses are mediated through RLKs by recognizing conserved pathogen-associated molecular patterns (PAMPs), induction of several members of these immune receptors may provide evidence for an “early warning system” initiated through pathogen recognition and provides the initial barrier that supports the host to deny pathogen establishment. Suppression of PTI response by some successful pathogen employing their pathogenic effectors encounters another additional layer of defense, facilitated by the R-genes^15,16^. Investigation revealed up-regulated expression of members of the NB-LRR containing R-proteins of the resistant host, VM84 might be playing a crucial role in conferring effector triggered immunity (ETI). Prompt induction of these R-genes activates the downstream immune machinery associated with the cell wall reinforcement, production of secondary metabolites, induction of oxidative stress and ROS^13,15^. The present findings advocate that although the different mode of defense arsenal displayed in response to MYMIV, the effective recognition of pathogenic *avr* gene product by the resistant host potentiated the innate immune response.

Plants respond to biotic stresses by activating hormonal signaling pathways that pursue to act either synergistically or antagonistically to shape the tangible defense response. Resistance to biotrophic pathogens is mostly an outcome of the SA-mediated signaling that operates antagonistically to suppress the Jasmonic Acid (JA) pathway^15,17^. In the present study, both KEGG enrichment and MapMan analyses emphasized a post-inoculation re-programming of the hormonally regulated genes. For example, core components of salicylic acid (SA)-signaling pathway including Phenylalanine ammonia lyase (PAL, EC 4.3.1.24) and non-expresser of pathogenesis related gene 1 (NPR1) were found to be highly up-regulated in VM84 with similar expression profiles. NPR1 acts as a cytosolic receptor of SA that interacts with TGA transcription factors (TFs) and activates pathogenesis related (PR) genes thereby providing the antiviral response^18^. However, significant induction of these SA-responsive genes was not observed in the MYMIV-inoculated susceptible host. Additionally, the induction of PAL, a key enzyme in SA biosynthesis suggests that SA biosynthesis in *V*. *mungo* takes place via the phenylalanine dependent pathway. Associated with the SA induced responses are hypersensitive response (HR) and systemic acquired resistance (SAR)^19^. PR proteins comprise of a class of antimicrobial peptides that are hallmarks of SAR and synthesized by the downstream activity of SA-induced activation of NPR1^20^. DEGs encoding, PR1, PR5 and PR10 of MYMIV-inoculated resistant host suggest that the SA-mediated signaling pathway was operative in providing immune response eliciting systemic acquired resistance (SAR).Despite the fact that jasmonic acid (JA) act antagonistically to SA in some species, expression of two DEGs related to JA and ethylene (ET)-signaling, lysyl oxidase (LOX) and 1-aminocyclopropane-1-carboxylate oxidase (ACC-oxidase) were also found to be altered with an up-regulated expression in the inoculated leaves of VM84 as also reported by Powell *et al*.^21^ and Jayaswall *et al*.^18^. Although endogenous SA levels were not quantified in the present study, yet the data obtained advocates the involvement of SA-dependent pathway is primarily operative in response to MYMIV which is in concurrence with our previous reports^4,5^.Generation of ROS and oxidative damage under pathogenic pressure is considered as an anti-microbial response that induces cell death and initiates HR at the site of attempted invasion^22,23^. Simultaneously, induction of huge quantities of free radicals is toxic to the host cell leading to oxidative stress. In response to MYMIV infection, expression of ROS scavenging enzymes that maintain a steady-state level of free radicals were observed to stimulated at various stages of pathogenesis and might be the reason for a robust immune reaction in VM84. Though the expression of most of these enzymes were found to be greater in VM84, almost all these genes were also differentially expressed compared to their mock controls in susceptible T9. In agreement with the previous reports^24,25^, the present study also demonstrated that a strong antioxidative defense mechanism was operative in response to MYMIV regardless of their background and a rapid detoxification of free radicals was critical for the survival of host plants under high disease pressure.

DEGs implicated in transcriptional regulation were significantly enriched in the leaves upon MYMIV inoculation. Several TFs were well represented within the dataset comprising of bHLH, WRKY, ZF-C2H2_,_ MYC, NAC, GATA, bZIP, WD-40 and AP2. WRKYs, one of the largest families of TF, have been shown to play significant rolesin response to pathogenic infection including viruses^26^. In particular, seven members of the WRKY gene family were up-regulated in VM84, of which, WRKY33 showed a constitutive induction in all the three time points with more than five-fold expression that might play a substantial role in MYMIV-resistance. Transcripts of 3 NAC TFs were also found to be amplified in response to MYMIV as previously demonstrated in rice following infection both by rice stripe virus and rice tungro spherical virus^27^. Other strongly expressed TFs include bHLH, MYB, GATA, ZF, bZIP noted during the immune response of *V*. *mungo* that were reported to be involved in augmenting tolerances against various pathogens including viruses^28^. Alterations in the MYMIV-responsive-TF repertoire are exceedingly critical for a rapid transcriptional reprogramming of downstream genes; thereby involved in the fine-tuning of immune response.

Cell wall provides a crucial line of physical barrier to pathogens that prevents pathogenic growth and proliferation^29,30^. A properly sealed cell wall can starve the pathogen from available nutrients and avert viral spread through plasmodesmata. Reinforcement of the cell wall by the activity of several enzymes including pectin acetyl esterase, pectin esterase and endoglucanases has been detected in the resistant host in response to the viral intruder. Lignin deposition in the cell wall is another way to restrict plant pathogens^31^. Elicitation of PAL and O-methyltransferase (OMT) upshots deposition of lignin by synthesizing precursor molecules that further strengthens the cell wall thereby playing prominent roles in immune responses. Similar functions were also observed for callose synthase and xyloglucanendo-transglycosylase that elicit cell wall fortification and restoration mechanisms. On the contrary, weaker induction of these enzymes in the susceptible background leads to compromised manifestation of sensitive reaction.

Plant ABC-transporters are involved in biotic and abiotic stress-responses that influence cellular detoxification processes^32^. These proteins are responsible for vacuolar sequestration of pathogen secreted toxic chemicals and thereby nullify the effect of the pathogen^33^. In the present study, elevated levels of ABC transporters as noted in VM84 following MYMIV-challenge, suggesting resistant genotype confiscates toxic, pathogenic metabolites into the apoplastic region and vacuoles calibrating the host’s regular metabolic activities.

Another crucial component of host defense is the synthesis of secondary metabolites upon pathogenic ingression. Pathway enrichment analysis mapped several transcriptsfor secondary metabolite biosynthesis involved in the production of aromatic amino acids, terpenoid, flavonoids, phenylpropanoids and phenolics. These compounds serve as precursor molecules in the syntheses of antimicrobials including phytoalexins and phytoanticipins which are directly involved in immune reaction against pathogens^34,35^. In the present study, elevated expression of 3 DEGs of the Cytochrome P450 monoxygenase family, CYP83, CYP710 and CYP51 were noted in VM84 suggesting their plausible roles in imparting resistance. Additionally, other phenylpropanoid biosynthetic genes such as 4-coumarate ligase (4CL) and caffeoyl-o-methyltransferase showed altered expression upon MYMIV-infection. As per speculation and followed by qPCR confirmation, phenylalanine ammonia lyase (PAL) was also induced in VM84, confirming its role in immune reaction against MYMIV infection.

The present study revealed some important mechanisms operating to tailor resistance against MYMIV. RNA silencing is one such mechanism that plays a critical role in host resistance particularly against viruses^36^. The Argonaute (AGO) proteins are vital components of RNA Induced Silencing Complexes (RISCs) associated with gene silencing via RNA interference either by translational inhibition or by degradation of RNA. Cassava Brown Streak Virus (CBSV) induced amplified expression of AGOproteins has been reported in cassava^28^; while it has been demonstrated that constitutive expression of two AGO proteins, AGO2 and AGO5 are essential for restricting PVX in *Arabidopsis*^37^. In agreement with this report, expression of 4AGO transcripts were induced suggesting that RNA silencing may restrict the proliferation of MYMIV from further proliferation. Additional support is provided by the induction of dicer like proteins (DCL) in the resistant host. DCL proteins cleave host specified long dsRNA molecules and initiate antiviral RNA silencing and possibly participating in the immune response of *V*. *mungo*.

Ubiquitination associated protein degradation is an important component of the host arsenal that perceive and transduce pathogenic signals leading to protein degradation via proteasome by tagging ubiquitin to targeted proteins^38,39^. Presence of E3 ubiquitin ligase, proteasome subunit beta, RING finger ligase amongst the DEGs in VM84 supports their involvement in degradation of viral proteins upon pathogenesis. While, one ubiquitin-conjugating enzyme was down-regulated in the MYMIV susceptible T9. Furthermore, induced accumulation of subtilisin proteases may lead to the stimulation of defense signaling pathways and immune priming^40^.

Heat-shock proteins (HSPs) constitute an important class of regulatory molecules that govern stress-induced stabilization, folding and denaturation of proteins. Several members of heat shock proteins including HSP70 with up-regulated expression were observed in VM84 assuming their involvement in the MYMIV-resistance presumably by increasing stability to the host proteins and denaturingthe viral proteins under MYMIV-stress.

Several plasma membrane localized ATPases including V type proton and Ca^++^ ATPases have been identified that maintain ionic homeostasis thereby suggesting their significance in MYMIV-defense. Fluctuations in the cytosolic Ca^2+^ concentration as a function of Ca^++^ ATPases may trigger host’s surveillance by activating Ca^2+^ sensors that initiate intricate signaling by transducing pathogenic signals. Upregulated expression of calmodulins (CaM), calcium binding protein, calmodulin-like proteins (CMLs) and calreticulins possibly provide immunity by activating Ca^2+^-dependent protein kinases (CDPKs) and other downstream effectors. A delay in the induction of the calcium-signaling mechanism in T9 was attributed possibly due to the lack ofan efficient pathogen sensing mechanism^41^. In addition, MAPKs are known to be important modulators of ETI that directly process pathogenic signals from the receptors and convey the message to downstream effectors. In a recent work we have shown the vital role of one such MAPK, *VmMAPK1*, which exhibited contrasting expression in the two genotypes^5^, and restricts MYMIV-proliferation in transgenic tobacco^41^.

It has been previously reported that plants respond to virus infection via genetic rearrangement^42^. Moreover, genetic instability via activity of the transposons may contribute to the immune response in plants. In the current study, induction of LTR retroelements in VM84 might be responsible for genetic reorganization and hence could be responsible for providing new locus linked to the immunity. Furthermore, alteration in the expression of histone deacetylase perhaps leads to chromatin remodeling that increases further transcription of defense genes and might have provided protection against MYMIV.

## Conclusion

High throughput RNA-Seq unveils a comprehensive scenario on the genome wide expression profiles of virus-responsive transcripts in both resistant and susceptible *V*. *mungo* genotypes that facilitated to figure out the key molecular determinants conferring MYMIV-resistance. Resistant VM84 genotype displayed a rapid and augmented immune reaction and responded to MYMIV through eliciting expression of pathogen responsive genes that barred the viral intruder from being established. Transcripts involved in pathogen recognition via PTI and ETI were more promptly and abundantly expressed in the resistant genotype when compared to the susceptible T9. Based on GO, KEGG and MAPMAN analyses, DEGs implicated in defense response such as ANK, DEF, SOD, RLKs, NAC and WRKY33, CAM were all operative in the resistant background that remained functionally involved in canonical pathways to biotic stresses, defense signaling, transcriptional regulation, antioxidant activity and biosynthesis of secondary metabolites. qPCR based time course expressional dynamics of these fate-determining candidate transcripts across the infection stages established their involvement in the antiviral response demonstrating a strong correlation between defense genes induction and enactment of immune responsive reaction. Moreover, induction of AGO, DCL and LTR retro-elements activate RNA-silencing and transposon mediated genome instability, respectively that advocates the involvement of non-canonical defense pathways. While not definitive, frail implementation of these responses through weaker induction of the above mentioned genes in the susceptible background resulted in a compromised immune reaction. These genomic resources identified through RNA-Seq study not only offer a strong scientific platform considering the impact of MYMIV-pathogenesis but definitely overcomes the infancy in the current understanding of the hosts responses to MYMIV. Therefore, the current investigation is of colossal importance in view of its potential of genetic resilience and will accelerate MYMIV-resistance breeding through genetic enhancement of *V*. *mungo* and other grain legumes.

## Materials and Methods

### Plant material, growth conditions and MYMIV inoculation

Two *V*. *mungo* genotypes showing contrasting reactions to MYMIV were selected for the present study. VM84 is a recombinant inbred, MYMIV-resistant line that harbours the MYMIV-resistance gene *CYR1*, while, T9 is used as the susceptible cultivar. VM84 was selected based on its good agronomic traits including plant architecture, overall yield and resistance to MYMIV while T9 is a widely grown, popular cultivar with high agronomic traits^43^. Disinfected seeds of both these genotypes (surface sterilized with 0.1% HgCl_2_ for 5 min and subsequently rinsed in distilled H_2_O) were sown in sterilized composite soil and grown in a glass house at 25 ± 2 °C, 16/8 h light/dark photoperiod (light intensity of 500 μmol m^−2^s^−1^) and 80% relative humidity.

Seedling growth was allowed until the first trifoliate leaves expanded completely (approx. 21 days post germination). Plants were forced inoculated with MYMIV using the trapper method as described by Kundu *et al*.^5^. Populations of white fly (*Bemisia tabaci*), the vector of MYMIV were collected from the insectary facility of the experimental farm of Bose Institute. Diseased plants, showing typical mosaic symptoms were used as the source of virus inoculum. The flies were virulified by forced feeding on symptomatic leaves for 24 h (acquisition access period) and then released onto healthy T9 and VM84 plants, allowing a 24 h inoculation access period to facilitate infection. Mock-inoculations were done using aviruliferous whiteflies. Thereafter, leaves of inoculated and mock samples were harvested at 3, 7 and 10 days post inoculation (dpi), snap-chilled in liquid nitrogen and stored at −80 °C until RNA isolation. Three independent biological replicates (5 plants each) were performed for each sampling time point. MYMIV-infectivity assays were performed as previously described by Kundu *et al*.^5^.

### Library construction and Illumina sequencing

Total RNA was extracted from the MYMIV-inoculated and mock-inoculated leaf samples for each time point using Trizol reagent (Invitrogen, CA) and purified using the RNeasy Plant Mini Kit (Qiagen, USA). Qualitative and quantitative estimation of the extracted RNA were done by 1% denaturing agarose gel electrophoresis and Agilent 2100 Bioanalyzer (RNA Nano Chip, Agilent, CA). An equimolar amount of RNA samples with an RNA integrity number (RIN) of 7 or more were pooled together for further analysis. About 20–30 µg of pooled RNA was sent to Genotypic Technologies Pvt. Ltd. (India), the commercial service provider for sequencing. RNA-Seq libraries were constructed using 4 μg of RNA following the IlluminaTruSeq RNA sample prep Kit v2 (Illumina Inc., CA). Sequencing was carried out using the Illumina platform as per manufacturer’s recommended protocols to generate 100 bp paired end reads (http://www.illumina.com/systems/hiseq_systems/hiseq_2000_1000/kits.ilmn).

### RNA-Seq data processing and quality control

Quality control of raw data was performed using the FastQC programme (http://www.bioinformatics.babraham.ac.uk/projects/fastqc/) to generate high quality reads. Raw reads were filtered for trimming adapter/primer sequences, removal of low quality reads and viral sequence if any. Adapter-free, good quality reads were submitted at NCBI in the Short Read Archive (SRA) database.

### De-novo transcriptome assembly and clustering

Quality controlled Illumina HiSeq reads of mock and inoculated samples of VM84 and T9 were de-novo assembled using Velvet 1.2.10 tool (http://www.ebi.ac.uk/zerbino/velvet/) to generate contigs. The assembled contigs were processed through Oases 0.2.08 (http://www.ebi.ac.uk/zerbino/oases/) pipeline to generate transcripts. Removal of redundant sequences was done with CD-HIT-EST run and subsequent clustering of non-redundant (nr) sequences using CD-HIT v 4.5.4 (http://www.bioinformatics.org/cd-hit/) with a sequence identity threshold of 80%.

### Analyses of differentially expressed genes (DEGs)

Differential gene expression analyses were accomplished using DESeq v 1.8.1 package (http://www-huber.embl.de/users/anders/DESeq/). Results were computed based upon a p-value ≤0.05 and expression value > 0. To identify statistically significant DEGs, fold change was computed using the log2 (fold change) ≥2 value as a threshold. Only Q-significant transcripts were considered for further analyses.

### Functional annotation, GO categorization and MapMan analysis

Due to lack of whole genome sequence information in *V*. *mungo*, functional annotations of the unannotated transcripts were carried out using the multiple Viridiplantae databases as target. Functional categorization of the transcripts was performed using BLAST2GO tool to segregate the transcripts into respective gene ontologies using controlled vocabularies to recover GO terms with their BLASTx description. Pathway mapping of the annotated transcripts was done using the KAAS server against the KEGG database (Kyoto Encyclopedia of Genes and Genomes) (http://www.genome.jp/kegg) to identify the involvement of DEGs in respective pathways. MapMan analysis was carried out by running the Mercator tool (http://mapman.gabipd.org/web/guest/mercator) and visualized as MapMan bins. Genes as per the DESeq output file with their respective Log2 fold changes were used as MapMan input to visualize the expression changes. A workflow of the experimental details have been presented in the Supplementary File 1.

### Quantitative real-time PCR analysis

Quantitative real-time PCR (qPCR) was performed from the same RNA samples used for NGS to validate the expression of 15 candidate DEGs. First strand cDNA synthesis was carried out from 1 μg total RNA from each time point of inoculated and mock samples of VM84 and T9 using the RevertAid first strand cDNA synthesis kit (Fermentas, Canada). Gene-specific primers were custom designed using Primer 3 Plus (http://frodo.wi.mit.edu/cgi-bin/primer3/primer3_www.cgi) with an expected amplicon size of 200–250 bp and verified with Oligocalculator (http://biotools.nubic.northwestern.edu/OligoCalc.html). The *V*. *mungo* Actin gene (ACT, GenBank Accession Number JZ078743) was used as an internal control^44^ to normalize the qPCR data. All the primer sequences were tabulated separately in the Supplementary File 2. Briefly, reactions were carried out in a 20 μl mixture containing 20 ng cDNA, 0.3 μM forward and reverse primers and 12.5 μl SYBR Green PCR Master Mix. qPCR cycling conditions were: an initial denaturation at 95 °C for 5 min, followed by 40 amplification cycles at 95 °C for 15 s, annealing at 50–55 °C for 30 s, and extension at 70 °C for 30 s. The relative expression of the target genes were calculated using the 2^−ΔΔCt^ method^45^. All qPCR reactions were processed in triplicates. Standard error (SE) was calculated for respective transcripts to compare data of MYMIV and mock-inoculated samples considering P < 0.05 as statistically significant.

## Supplementary information

Supplementary Information

## Data Availability

The datasets generated during the current study are available in the SRA repository as under: • http://www.ncbi.nlm.nih.gov/sra/SRX1082731: Transcriptome Library of *Vigna mungo* RIL VM84 infected with MYMIV. • http://www.ncbi.nlm.nih.gov/sra/SRX1032950: Transcriptome Library of mock inoculated *Vigna mungo* RIL VM84. • http://www.ncbi.nlm.nih.gov/sra/SRX1058327: Transcriptome Library of *Vigna mungo* RIL Cv. T9 infected with MYMIV. • http://www.ncbi.nlm.nih.gov/sra/SRX1058325: Transcriptome Library of mock inoculated *Vigna mungo* Cv. T9.
