# Supplementary material for: Complex molecular mechanisms underlying MYMIV-resistance in Vigna mungo revealed by comparative transcriptome profiling
Source: Sci Rep. 2019 Jun 20;9:8858. doi: 10.1038/s41598-019-45383-w (PMC6586629; doi:10.1038/s41598-019-45383-w)
Supplement: Supplementary file 1 — Supplementary Information [file 41598_2019_45383_MOESM1_ESM.pdf]

Complex molecular mechanisms underlying MYMIV-resistance in *Vigna mungo* revealed by comparative transcriptome profiling

Anirban Kundu[1,2*], Pankaj Kumar Singh[1*], Avishek Dey[1], Sayak Ganguli [3] and Amita Pal**[1]

[1]Division of Plant Biology, Bose Institute, Kolkata-700054, India.
[2]Ramakrishna Mission Vivekananda Centenary College, Rahara, Kolkata- 7000118, India.
[3]Theoretical and Computational Biology, AIIST, Palta, Kolkata, India.

* Both the authors contributed equally
** Corresponding Author: amita@jcbose.ac.in

Supplementary Figure S1: NGS workflow blueprint for the RNA sequencing data.

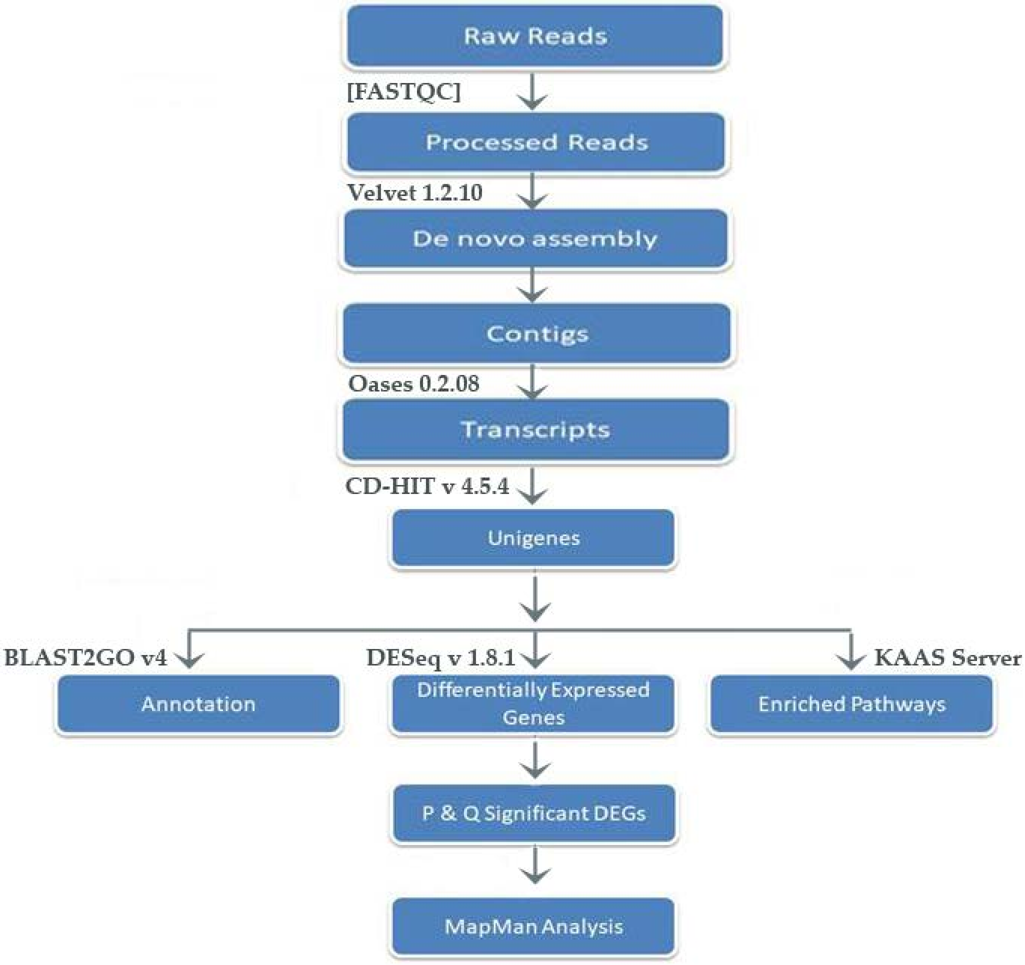

Supplementary Table S2: Primers list of transcripts used for qPCR analyses. Sequence information and amplicon characteristics of primers of 15 candidate genes used for qPCR expression analyses.

Sl. No.	Name	Sequence (5` to 3`)		Amplicon Length (bp)
1.	Calmodulin (CAM)	F	CGAAGAATGCCACAACATGA	237
		R	CTACTCAGGGCGATTGAAC	
2.	Heat shock protein 70 (HSP70)	F	TTCAAACCCTCCTTGGGACAC	203
		R	GAATGAAAGCTGGCCAGAAG	
3.	ABC transporter (ABC)	F	CAGGCCTATGTGACTCAAG	180
		R	ACTCTCCTCTTCTGTCCTCC	
4.	NAC transcription factor (NAC)	F	GTGGAGGGTGTGAAGGTTATC	224
		R	CTCCGTCTCAGGTTCCCATGG	
5.	Defensin (DEF)	F	GTGGCTCTGAGACTCACATG	166
		R	CCGGTAAGCCTTCTCCACGC	
6.	Argonaute (AGO)	F	GACGTTGTCTCTGCTGGCAG	250
		R	ACACCTCCTCCTACATCAGC	
7.	Ankyrin (ANK)	F	TACCACCCGTTGCACATAGC	209
		R	GCAGGCAAGTACAACCCATC	
8.	E3- Ubiquitin ligase(E3 UBL)	F	GTTTCGATATGCTTCAGGC	139
		R	AGAGCTGCCTGAATGAAATG	
9.	R-gene (NB-LRR)	F	CATGGGCTGGAACACCTCC	236
		R	GCTCTCTATTCAGTGTCTTGAC	
10.	Superoxide dismutase (SOD)	F	CCCTTGTCTGGGGAGGTTAT	209
		R	GCACATCACACCATCCAAGGT	
11.	WRKY transcription factor (WRKY33)	F	GATCACGAATCTCTCTCAGG	238
		R	CGCTGGACCCCTCTAACAC	
12.	Thioredoxin (TRX)	F	GAATCACTCCTCTGCGGTTC	168
		R	TCCATAGCGGGAGAGATGAG	
13.	Peroxidase (PER)	F	CTTGGCACAAAGCCTAGCTC	164
		R	GAGCAGCCCAATATACTCGC	
14.	Cysteine protease (CSP)	F	TCCTGCAAATTTGATAAAAGCA	190
		R	ATGGCATGAGACACCACCA	
15.	Pathogenesis related protein 1 (PR1)	F	CTGGCAAAGCCAAGAGTGAT	248
		R	AGCTCTCACAATTATGCAGC	
